# Modulation of HJURP (Holliday Junction-Recognizing Protein) Levels Is Correlated with Glioblastoma Cells Survival

**DOI:** 10.1371/journal.pone.0062200

**Published:** 2013-04-25

**Authors:** Valeria Valente, Rodolfo Bortolozo Serafim, Leonardo Cesar de Oliveira, Fernando Soares Adorni, Raul Torrieri, Daniela Pretti da Cunha Tirapelli, Enilza Maria Espreafico, Sueli Mieko Oba-Shinjo, Suely Kazue Nagahashi Marie, Maria Luisa Paçó-Larson, Carlos Gilberto Carlotti

**Affiliations:** 1 Department of Clinical Analysis, Faculty of Pharmaceutical Sciences of Araraquara, University of São Paulo State (UNESP), Araraquara, Brazil; 2 Center for Integrative Systems Biology (CISBi), NAP-USP, Ribeirão Preto, Brazil; 3 Molecular Oncology Research Center, Barretos Cancer Hospital, Barretos, Brazil; 4 Department of Surgery and Anatomy, Faculty of Medicine of Ribeirão Preto, University of São Paulo (USP), Ribeirão Preto, Brazil; 5 Department of Cellular and Molecular Biology, Faculty of Medicine of Ribeirão Preto, University of São Paulo (USP), Ribeirão Preto, Brazil; 6 Department of Neurology, Faculty of Medicine, University of São Paulo (USP), São Paulo, Brazil; NIH/NCI, United States of America

## Abstract

**Background:**

Diffuse astrocytomas are the most common type of primary brain cancer in adults. They present a wide variation in differentiation and aggressiveness, being classified into three grades: low-grade diffuse astrocytoma (grade II), anaplastic astrocytoma (grade III) and glioblastoma multiforme (grade IV), the most frequent and the major lethal type. Recent studies have highlighted the molecular heterogeneity of astrocytomas and demonstrated that large-scale analysis of gene expression could help in their classification and treatment. In this context, we previously demonstrated that HJURP, a novel protein involved in the repair of DNA double-strand breaks, is highly overexpressed in glioblastoma.

**Methodology/Principal Findings:**

Here we show that HJURP is remarkably overexpressed in a cohort composed of 40 patients with different grade astrocytomas. We also observed that tumors presenting the higher expression levels of HJURP are associated with poor survival prognosis, indicating HJURP overexpression as an independent prognostic factor of death risk for astrocytoma patients. More importantly, we found that HJURP knockdown strongly affects the maintenance of glioblastoma cells in a selective manner. Glioblastoma cells showed remarkable cell cycle arrest and premature senescence that culminated in elevated levels of cell death, differently from non-tumoral cells that were minimally affected.

**Conclusions:**

These data suggest that HJURP has an important role in the maintenance of extremely proliferative cells of high-grade gliomas and point to HJURP as a potential therapeutic target for the development of novel treatments for glioma patients.

## Introduction

Gliomas are the most frequent type of primary brain cancer in adults and encompass a spectrum of tumors varying in differentiation and aggressiveness. However, nearly all low-grade tumors eventually progress to high-grade malignancies. Astrocytomas, which are glial tumors composed of cells resembling astrocytes, account for more than 60% of the cases of glioma [1]. According to histopathological features, such as level of anaplasia, mitotic index, cellularity, microvascular proliferation and presence of necrosis, adult astrocytomas are classified into three types: low-grade diffuse astrocytoma (grade II), anaplastic astrocytoma (grade III) and glioblastoma multiforme (grade IV) [2]. Among them, glioblastoma multiforme (GBM) is the most frequent and the major lethal type of brain cancer. These tumors are extremely proliferative, invasive and highly vascularized, characteristics that lead to a mean survival time of 1 year for affected patients [1], [3]. Due to the ineffectiveness of currently available treatments, which results from the difficulty of achieving complete resection and the resistance of tumor cells to chemo and radiotherapy, the need for novel therapeutic targets for GBM treatment becomes urgent.

Recent studies have highlighted the heterogeneity of gliomas and demonstrated that molecular and genetic analysis could help in their classification and in the design of treatment protocols. Microarray-based expression profiling has characterized molecular subtypes of brain tumors related with distinct malignancy grades and clinical prognosis [4], [5], [6], [7], [8]. Two genes have been shown to be especially robust biomarkers of glioma prognosis - methylguanine-DNA-methyltransferase (MGMT) [9] and isocitrate dehydrogenase 1 (IDH1) [10], [11]. Hypo-methylation of the MGMT promoter is correlated with glioblastoma resistance to temozolomide (TMZ) chemotherapy, and consequently with worse prognosis, due to the reduction in TMZ induced alkylation when MGMT is overexpressed [9]. More recently, a 4-gene signature, highly correlated with survival of glioma patients, was identified through a cross-validation approach. From this study an optimized risk-score model was validated. The biomarkers identified in this study were *CHAF1B* (chromatin assembly factor 1), *PDLIM4* (LIM domain gene), *EDNRB* (endothelin receptor type B) and *HJURP* (Holiday Junction Recognizing Protein). EDNRB overexpression is associated with better prognosis conferring longer survival to patients. The overexpression of the other three genes is correlated with a higher risk of death [12].

HJURP is a novel protein recently shown to be required for CENP-A loading in the centromeric chromatin and for the assembly of functional kinetochores [13], [14], [15]. Also, Kato and collaborators (2007) have demonstrated that HJURP is overexpressed after DNA damage induction, interacts with components of the DNA repair machinery and acts in homologous recombination, suggesting a possible relevance for HJURP in DNA double-strand breaks (DSB) restoration [16], [17]. Additionally, it was observed that HJURP expression levels are increased in the majority of lung and breast cancers and correlate with poor survival prognosis [16], [18]. We previously demonstrated that HJURP is highly overexpressed in GBM [19]. Here we found that HJURP over expression in astrocytoma patients of our cohort is also associated with poor survival. Furthermore, we demonstrated that HJURP knockdown in different cell lines significantly affected the survival of glioblastoma cells but did not impact non-tumoral cells. After HJURP silencing, T98G and U87MG cells showed cell cycle arrest and premature senescence, respectively, which culminated in elevated levels of cell death for both cell lines. These findings support the hypothesis that HJURP might have an important role in the progression and/or maintenance of malignant gliomas, and possibly represent a novel target for development of new therapies for glioma patients.

## Results

### HJURP is Highly Over Expressed in Malignant Gliomas and its Expression Levels are Inversely Correlated with Patient Survival

**Figure 1:**
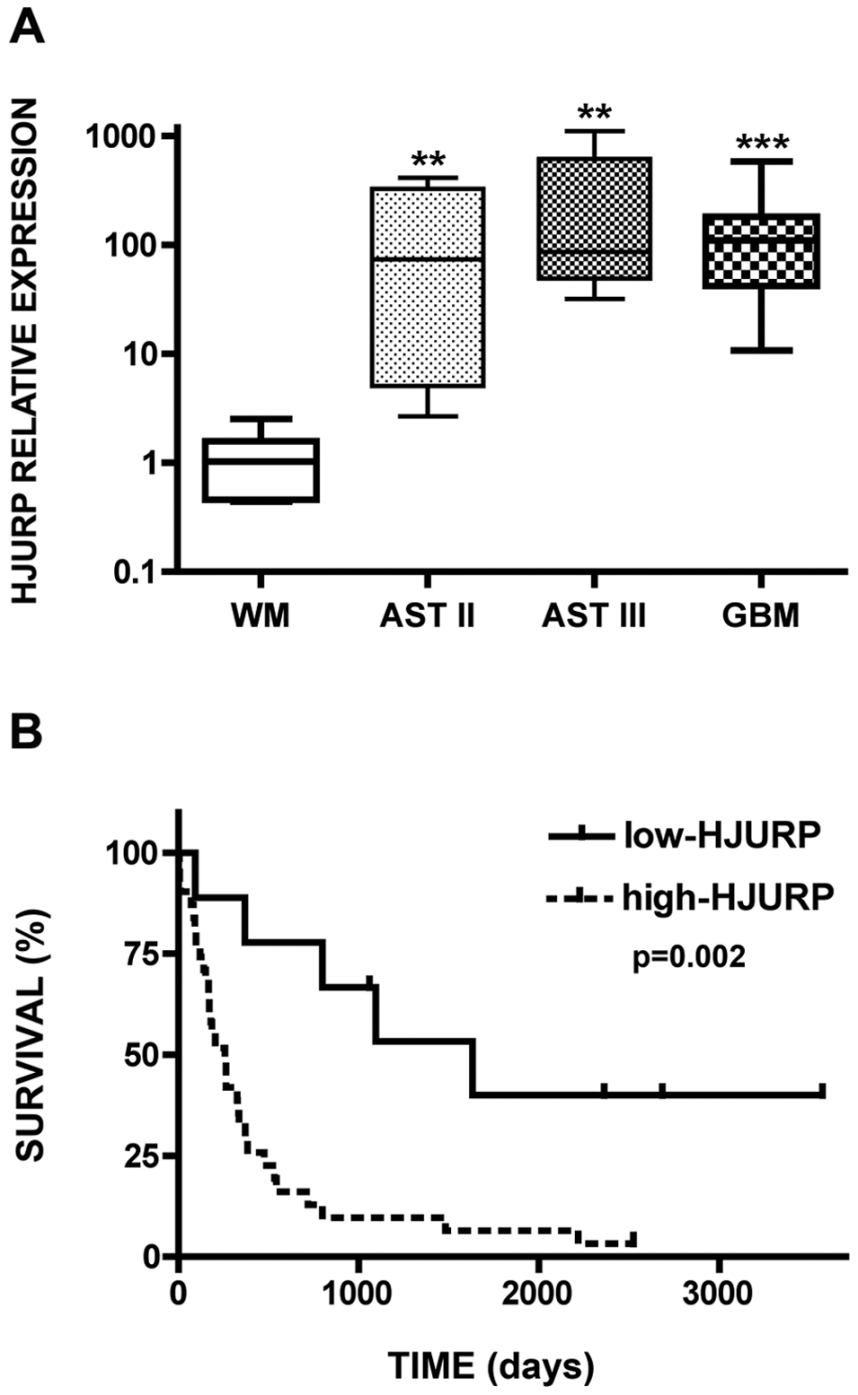
Levels of HJURP mRNA are increased in different grade astrocytomas and correlated with survival prognosis. (A) Expression levels of HJURP mRNA in samples of normal white matter (WM, n = 7), diffuse atrocytoma (AST II, n = 5), anaplastic astrocytoma (AST III, n = 5) and glioblastoma multiforme (GBM, n = 30) were evaluated by quantitative RT-PCR. Boxes represent lower and upper quartiles of HJURP relative expression ranges, with medians indicated. Whiskers represent the 10^th^ and 90^th^ percentiles. **P-value = 0.0025 and ***P-value <0.0001 in comparison with normal white matter, Mann-Whitney test. (B) Kaplan Meier survival curves for glioma patients according to the HJURP expression levels in the tumors. Patients were divided in two groups: i) HJURP relative quantities below 39.7 (threshold value determined by ROC curve) (solid line, n = 9), and ii) HJURP relative quantities above 39.7 (dashed line, n = 31). The P-value shown was obtained from a long-rank test. Graphs were plotted with GraphPad Prism 4.0 software.

We previously demonstrated that HJURP is highly over expressed in glioblastoma multiforme [19]. Here we show that HJURP is also over expressed in low-grade diffuse (grade II) and anaplastic (grade III) astrocytomas. HJURP mRNA was detected in extremely high levels in the majority of these tumors when compared with non-tumor white matter (Figure 1A, Table S1). Median values of the relative expression of HJURP mRNA were 74 and 86 in astrocytomas of grade II and III, respectively. Amongst the five cases of low-grade diffuse astrocytomas analyzed, four showed HJURP amounts varying from 7.5 to 413.1, which represent more than 3.9 fold the median value of normal white matter, which varied from 0.44 to 2.53. The anaplastic astrocytomas revealed even higher quantities of HJURP mRNA. All grade III tumors analyzed showed more than 16 fold the amounts of HJURP mRNA of normal white matter, with relative quantities varying from 31.9 to 1105 in these tumors. In GBM samples HJURP is also consistently over expressed, in 28 out of 30 cases evaluated, HJURP mRNA was detected in levels at least 13 fold greater than that of normal white matter, with relative expression varying from 25 to 583 approximately (Table S1). Interestingly, we also observed that different ranges of HJURP relative expression (RE) are correlated with patient survival. Patients that developed tumors presenting HJURP RE <100 survived for 31 months on average, while patients presenting tumors with HJURP RE between 100–250 or >250 survived for 16.4 or 5.7 months on average, respectively (Table 1). Indeed, statistical analysis revealed that patients with survival periods shorter than 18 months presented significantly (p = 0.02) higher levels of HJURP when compared to those showing longer life span (data not shown).

**Table 1:** HJURP expression ranges are correlated with patient survival.

HJURP Relative Expression	Mean Survival (months)	n°Cases
0–100	31.13	18
100–250	16.44	16
>250	5.57	6

P-value = 0.002, long-rank (Mantel-Cox) test.

To further investigate HJURP as a predictive factor of patient survival, we defined groups with low and high HJURP expression using the ROC curve method and performed Kaplan Meier statistical analysis. The results revealed that patients in the high-HJURP group presented significantly worse survival prognosis than those from the low-HJURP group (Figure 1B). Individuals suffering with tumors expressing higher levels of HJURP (RE >39.7, n = 31) showed a mean survival period of 16 months, while individuals whose tumors presented lower amounts of HJURP (RE <39.7, n = 9) showed a mean survival period of 45 months (Table S1). To strength our observation of HJURP as a predictive variable of survival prognosis, we performed a meta-analysis with 3 different expression datasets of glioma samples: the Repository of Molecular Brain Neoplasia DaTa (REMBRANDT), The Cancer Genome Atlas (TCGA) and two publically available microarray experiments (GSE4271 and GSE4412). The results obtained in the analyses with the three datasets showed a significant correlation between HJURP levels and patient survival (Figure S1).

To evaluate the HJURP potential as an independent predictive factor of survival prognosis we also performed a multivariate analysis including the available clinical variables (patient age and tumor grade), with our results and with the public microarray data GSE4271 and GSE4412 that included tumor samples of different grades. HJURP expression level was an indicator of poor survival prognosis in both datasets: our qRT-PCR data (hazard ratio = 3.48; p = 0.02) and the public microarray data (hazard ratio = 2.38; p = 0.001). Higher-grade tumors also showed a significant correlation with worse survival prognosis in the analysis of public data when microarray data were combined (hazard ratio = 2.41; p = 0.006) or evaluated separately (GSE4271, hazard ratio = 5.07; p = 0.047; GSE4412, hazard ratio = 3.21; p = 0.001). In our sample set tumor grade did not correlate with survival prognosis, probably due to the small number of low-grade tumors in our cohort (Table 2, Table S1). These data reveal that *HJURP* expression level alone gives a strong risk score associated with prognosis of glioma patients.

**Table 2:** Multivariate analysis of prognostic factors in patients with glioma using Cox regression.

	qRT-PCR dataset		Microarray dataset (GSE4271+GSE4412)	
Factor	Hazard ratio (95% CI)	P value	Hazard ratio (95% CI)	P value
HJURP expression	3.48	0.02	2.38	0.001
Age	1.02	0.73	0.99	0.264
Tumor grade	–	0.667	2.41	0.006

### Knockdown of HJURP Affects the Viability of Glioblastoma Cells

**Figure 2:**
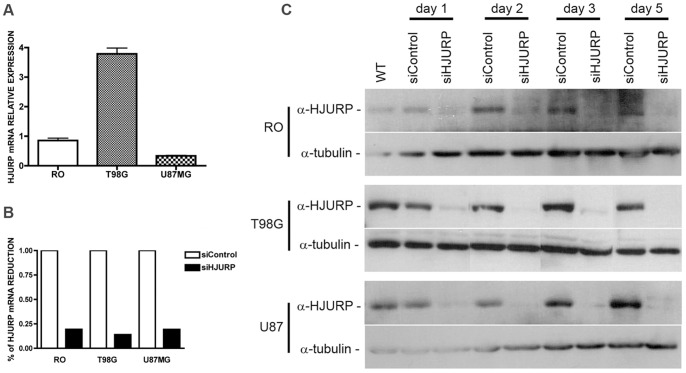
HJURP expression is remarkably reduced in cultured cells upon treatment with double stranded siRNA. (A) The HJURP mRNA levels for the non-tumoral cell RO and both tumoral cell lines, T98G and U87MG, were determined by qRT-PCR. (B) Indicated cell lines were transfected with control (scrambled) double-stranded siRNA (siControl) or with siRNA directed to HJURP mRNA (siHJURP). The HJURP mRNA levels were determined by qRT-PCR at the second day (48 h) after transfection. (C) Protein extracts of cell cultures without treatment (WT) or tranfected with the indicated siRNAs were collected at the first, second, third and fifty days post-transfection. Expression of HJURP protein was evaluated by immunoblot.

**Figure 3:**
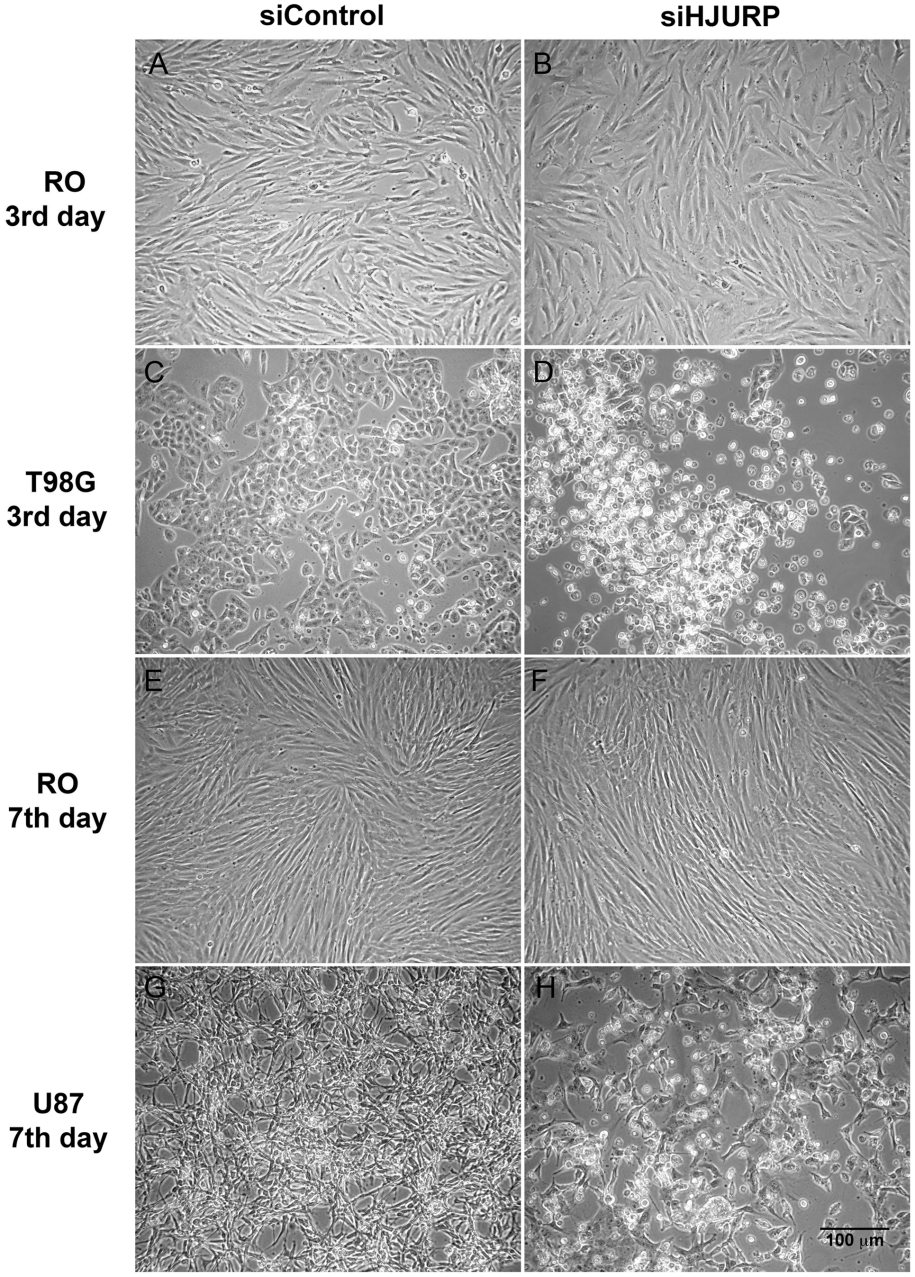
T98G and U87MG cells are dramatically affected by HJURP knockdown. Images of RO fibroblasts, T98G, and U87MG GBM cells, at the indicated times after transfection with siControl or siHJURP double-stranded RNAs, were captured under phase-contrast microscopy (Leica MC OS).

To investigate the role of HJURP in glioblastoma cells, we performed knockdown experiments using double-stranded synthetic RNA oligonucleotides (siRNAs) directed to the coding region of HJURP mRNA. We transiently transfected three cell lines, RO (non-tumoral fibroblast), T98G and U87MG (GBM cell lines), which express different levels of HJURP. The amounts of HJURP mRNA measured in T98G cells were 4.4 and 11.3 fold the quantities detected in RO and U87MG cells, respectively (Figure 2A). After 48 hours of transfection, the HJURP expression was significantly reduced at both the mRNA (Figure 2B) and protein (Figure 2C) levels in the three cell lines utilized, with a maximum HJURP knockdown (∼90%) between the third and the fifth day post-transfection. After this period there was a slight recovery of HJURP expression but a sizeable knockdown lasted until the seventh day post-transfection for RO and U87MG cell lines (data not shown). Interestingly, we noticed that HJURP silencing induced a dramatic effect on the maintenance of T98G and U87MG cells, while non-tumoral RO cells were minimally affected (Figure 3). Additional analysis of HJURP silencing in other non-tumoral cell line, HDPC, also showed no significant effect on cell morphology until the 7^th^ day post-transfection (Figure S3A–B). After three days of HJURP knockdown the majority of T98G cells were detached, having a rounded morphology (Figure 3C–D), while non-tumoral control cells were not morphologically altered (Figure 3A–B, Figure S3B). For U87MG cells similar alterations were observed after a longer period of HJURP depletion. Detached and small rounded cells were seen at the seventh day post-transfection (Figure 3G–H), while control cells were not apparently affected up to this time (Figure 3E–F, Figure S3B). On the basis of these morphology alterations, HJURP knockdown seems to induce cell death in both tumoral cells.

### Glioblastoma Cells Show Cell Cycle Arrest and Increased Apoptosis after HJURP Knockdown

**Figure 4:**
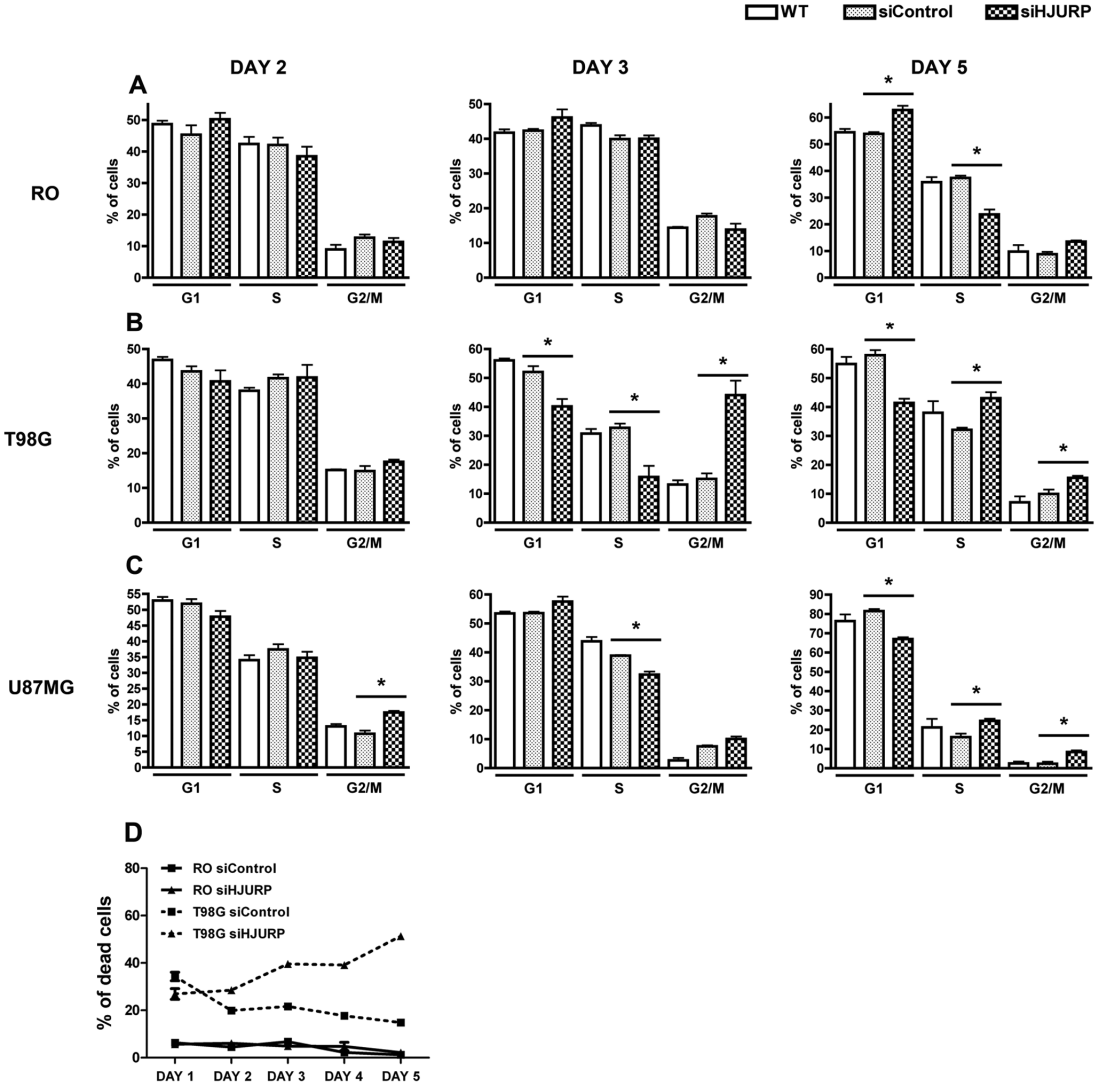
Cell cycle and viability analysis of non-tumoral and glioma cells after HJURP silencing. RO, T98G, and U87MG cells, were transfected with control double-stranded siRNA or with siRNA directed against HJURP mRNA. Cells submitted to the different treatments were fixed, labeled with propidium iodide and their DNA content was measured by flow cytometry analysis. (A, B and C) Percentage of cells in each cell cycle phase at different times after transfection. N = 3, *P<0.05, T test. RO and T98G cells subjected to the different treatments were stained with propidium iodide for viability analysis. (D) Percentages of cell death in 5 consecutive days after transfection are shown. n = 3, P<0.001 (T-test) for comparisons between T98G cells treated with siControl *versus* siHJURP at the third, fourth and fifth days.

The reduction of HJURP expression in T98G cells resulted in prominent cell cycle arrest shortly after knockdown was triggered. The percentage of T98G cells in G2/M phases of the cell cycle increased from approximately 15% up to 44% at the third day after HJURP silencing (Figure 4B, Figure S2). These cells were unable to escape the cell cycle arrest and also showed a significant increase in mortality rates that progressively elevated from the second day of siRNA treatment, reaching 51.3% at the fifth day post-transfection (Figure 4D). Non-tumoral cells showed only a slight but significant increment in the number of cells in G1 phase and a correspondent decrease in S phase cells after 5 days of HJURP knockdown (Figure 4A, Figure S2). In addition, the mortality rates of non-tumoral RO and HDPC cells treated with HJURP siRNA were similar to that of cells treated with control siRNAs (Figure 4D, Figure S3C).

**Figure 5:**
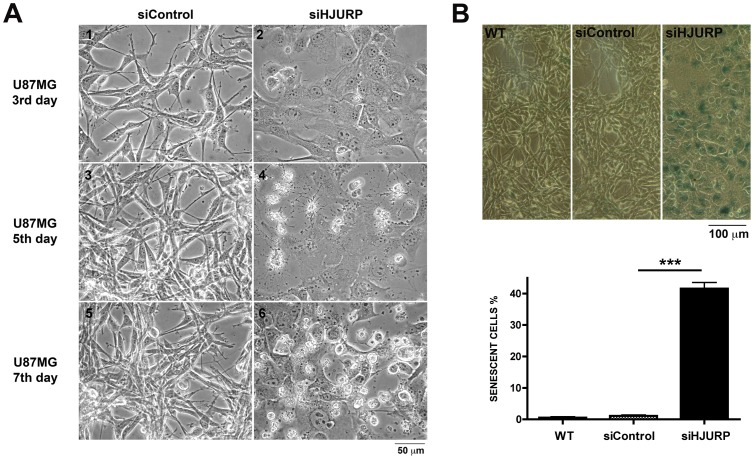
U87MG cells enter in premature senescence after HJURP knockdown. (A) Images of U87MG cells at the indicated times after transfection with siControl or siHJURP double-stranded RNAs were captured under phase-contrast microscopy (Leica MC OS). (B) U87MG cells subjected to the indicated treatments were processed for the β-galactosidase assay at the fifth day after transfection. Graphic shows the percentage of senescent cells in each condition. Measurements were performed in three independent experiments. ***P<0.0001, T-test.

U87MG cells were affected in a different manner by HJURP knockdown. Major differences were observed in the 5^th^ day after silencing, with a reduction in the number of cells in G1 phase and an increase in the number of cells in S and G2/M phases (Figure 4C, Figure S2). These cells also showed noticeable morphological changes that appeared gradually from the second day of siRNA treatment. U87MG cells, which are usually elongated and grow loosely attached as a sparse network (Figure 5A-1, A-3 and A-5), become flat, and spread out when HJURP levels are diminished (Figure 5A-2). These morphological alterations strongly suggested the occurrence of premature senescence. To further investigate this possibility we performed senescence associated β-galactosidase (SA-β-Gal) assay. U87MG β-Gal positive cells were initially detected after three days of HJURP knockdown and increased dramatically (up to ∼37 fold) after five days (Figure 5B, Table S2). SA-β-Gal analysis showed that RO and T98G cells did not present an increase in senescence after HJURP silencing (Figure S4, Table S2).

**Figure 6:**
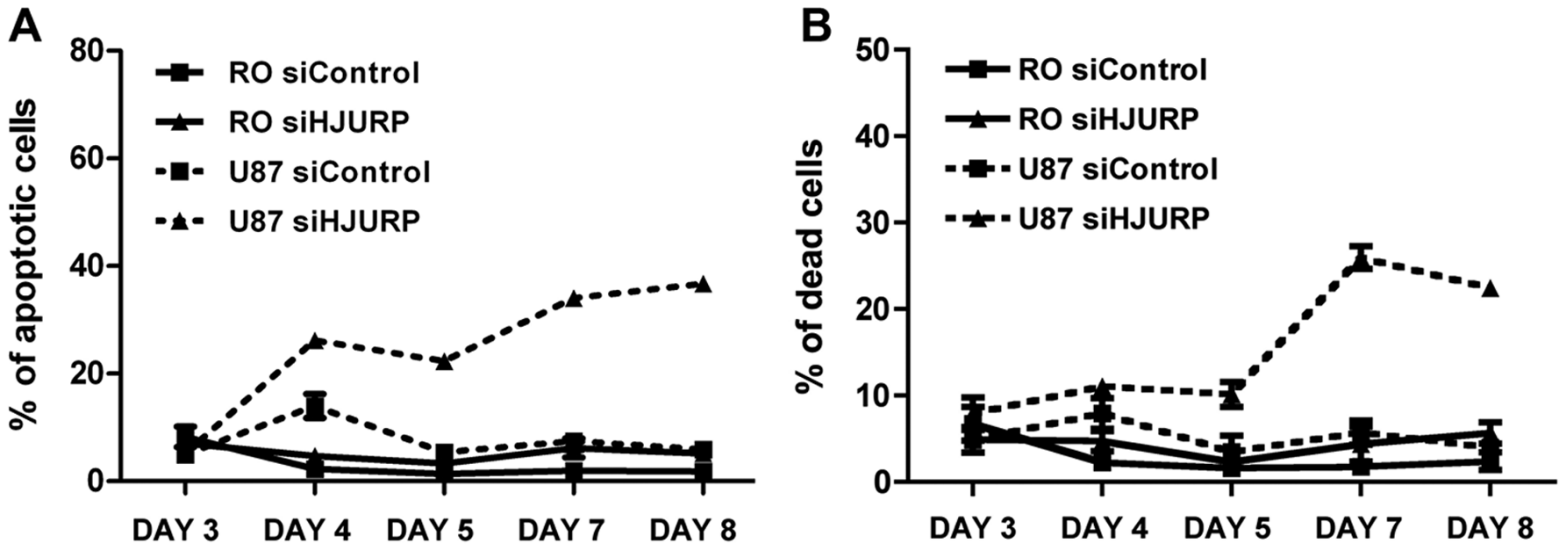
U87MG cells present decreased viability after HJURP silencing. U87MG cells were transfected with control double-stranded siRNA or with siRNA directed against HJURP mRNA. RO or U87MG cells subjected to the different treatments were processed for flow cytometer analysis with annexin V or propidium iodide labeling. Apoptotic (A) or dead (B) cells were quantified by flow cytometry (∼5,000 events) at the indicated days after transfection. N = 3, P<0.01 for comparisons between U87MG cells treated with siControl *versus* siHJURP from the fourth and fifth days after transfection in the analysis of apoptosis (A) or cell death (B), respectively.

After five days of HJURP depletion many U87MG cells also presented membrane blebs (Figure 5A-4) and at the seventh day a significant proportion of cells were round, shrunk and detached (Figure 5A-6). Accordingly, apoptosis levels progressively increased from approximately 5%, observed at the third day after transfection, up to 37% at the eighth day of HJURP silencing (Figure 6A). The elevated rates of apoptosis ultimately led to an abrupt increase in the percentage of dead cells at the seventh day post-transfection with HJURP siRNAs (Figure 6B). The viability of RO and HDPC non-tumoral cells was not significantly affected by HJURP knockdown during the period evaluated (Figure 6B, Figure S3C).

## Discussion

We have shown here that HJURP is highly over expressed in different grade gliomas encompassing low-grade diffuse and anaplastic astrocytomas, and GBMs (Figure 1A). The relative quantities of HJURP presented a broad variation among the analyzed tumors, from 7.5 to 1105, which inversely correlated with the patient’s overall survival (Figure 1B, Table S1). These data reinforce the hypothesis that HJURP is an independent prognostic factor of death risk for cancer patients. In agreement, published data has previously shown that HJURP over expression is correlated with diminished survival of lung and breast cancer patients [16], [18]. HJURP over expression was also included in a 4-gene signature associated with poor clinical outcome of high-grade malignancy gliomas [12]. Here, we show that HJURP can be used as an independent factor of survival prognosis based on the analysis of our results and public available data (Table 2). More aggressive tumors are highly resistant to therapies and susceptible to relapse. Since HJURP might be involved in DSB restoration by homologous recombination end joining, we suppose that the extremely elevated HJURP levels observed in the most aggressive tumors could be associated with the higher resistance to ionizing radiation therapy. Although, it has been reported that elevated levels of HJURP are associated with higher sensitivity to radiation in breast cancer cells [18], it is known that protein function can vary significantly in different cell types. This hypothesis is corroborated by the demonstration that HJURP stability is regulated through phosphorylation by the ATM kinase pathway [16]. The modulation of ATM expression was reported to be associated with radiosensitivity of U87MG cells. It was shown that demethylation of ATM promoter increased the levels of ATM protein and decreased the radiosensitivety of U87MG cells [20]. Thus, HJURP may act as the mediator of this response conferring higher resistance to ionizing radiation for the aggressive tumors in which their levels are extremely elevated. Further experiments must be done to investigate the function of HJURP in the radioresistance of glioma cells.

We also observed that HJURP knockdown strongly affects the maintenance of glioblastoma cells in a selective manner. After HJURP knockdown, T98G cells showed a remarkable cell cycle arrest at the third day of silencing (Figure 4B) and U87MG cells entered in premature senescence (Figure 5B). Previous studies have demonstrated that CENP-A and HJURP knockdown in primary fibroblasts elicits premature senescence in a p53 dependent manner [21], [22]. Once T98G cells harbor a mutation in p53, while U87MG cells are wild type, a reduction in CENP-A deposition, due to HJURP knockdown, could explain the different phenotype of these cells. Despite the differences in the behavior of T98G and U87MG cells, both presented elevated cell death levels after HJURP silencing (Figure 4D and 6) suggesting that HJURP is critical for different pathways of cell cycle progression.

Interestingly, while both tumor cells showed high cell death, the viability of non-tumoral cells were not significantly affected after HJURP silencing (Figure 4D, 6 and S3), which indicates that the maintenance of HJURP levels is more critical for the highly proliferating GBM cells than for control cells. In fact, T98G and U87MG cells present a doubling time approximately 2.6 fold shorter than HDPC and RO cells (data not shown). Thus, control cells undergo fewer divisions than GBM cells during the period of HJURP knockdown and possibly do not have CENP-A completely depleted at the centromeres. This could explain the absence of a significant effect in the viability of control cells subjected to HJURP silencing.

We might also speculate that the requirement of large amounts of HJURP is probably related to the genomic instability of cancer cells. Tumor cells are characterized by an accumulation of genetic alterations that drive tumorigenesis, including copy number variations, chromosomal rearrangements, point mutations and small insertions and deletions [23], [24], [25]. Recently, a comparison of the genome of lymphoblastoid cells and breast tumor cells isolated from the same patient has shown that structural variations in DNA are much more frequent in tumor than in non-tumoral cells. The breast cancer cell presented 94 structural variations including translocations, deletions, inversions and duplications, while the lymphoblastoid cell showed only four DNA structural alterations [26]. In addition, it was recently demonstrated that stringent regulation of HJURP is essential for genome stability, since its overexpression caused severe mitotic defects in HeLa cells [17]. Hence, we can hypothesize that HJURP is involved in the maintenance of more efficient DNA repair mechanisms, which are crucial for the tumoral cells to continue proliferating without collapsing due to the massive genomic instability. Altogether, these data suggest that the high levels of HJURP might have an essential function for survival of the extremely proliferative cells of high-grade malignancy gliomas and points out HJURP as a potential novel therapeutic target.

## Materials and Methods

### Ethics Statement

All patients involved in this study provided written consent for the use of tissue samples for research proposals. The consent procedure and research investigation performed with the human samples were approved by the Ethics Committee of the Faculty of Medicine of Ribeirão Preto – USP (HCRP #7645/99). All clinical procedures were conducted according to the principals of the Declaration of Helsinki.

### Tissue Samples and Cell Lines

Glioma samples were obtained from 40 patients submitted to surgical resection for tumor ablation at the Clinical Hospital of the Faculty of Medicine of Ribeirão Preto, University of São Paulo. The total set analyzed consisted of 5 diffuse astrocytomas, 5 anaplastic astrocytomas and 30 GBMs. The tumor grade was determined according to WHO criteria [2]. Non-neoplastic white matter samples (n = 7) were obtained from patients undergoing temporal lobectomy for epilepsy treatment. Tumors and non-neoplastic surgical samples were sectioned and snap-frozen in liquid nitrogen immediately after surgical removal. All tissue samples were microdissected for exclusion of areas presenting necrosis or not corresponding to the neoplastic diagnosis. Standardized conditions of storage and microdissection of tumor samples are important steps to guarantee reliability of data and the conclusions derived from them, especially for GBM, which are heterogeneous tumors often presenting necrosis [2]. The glioblastoma cell lines utilized (T98G and U87MG) are commercially available and were obtained from ATCC collection. As non-neoplastic control cells we used RO and HDPC primary cultures, which are fibroblasts isolated from skin and human dental pulp, respectively. RO cells were isolated from the prepuce skin of a 23 years old healthy male in 2005 in the laboratory of Dr. E. Espreafico. These cells are cultivated in standard conditions with Dulbecco’s modified Eagle’s medium (DMEM, Gibco), supplemented with 10% of fetal bovine serum and 100 U/mL penicillin, 0.1 mg/mL streptomycin and 0.25 µg/mL amphotericin B (Sigma-Aldrich). HDPCs were isolated from dental pulp of a 5 years old boy in the laboratory of Dr. C. Costa and Dr. J. Henbling. These cells have been cultivated for 1 year in the standard conditions with Minimum Essential Medium Eagle (α-MEM, Sigma-Aldrich), supplemented with 10% of fetal bovine serum and 100 U/mL penicillin and 0.23 mg/mL streptomycin (Gibco).

### Cell Culture and Transfection

All cell lines were cultivated in DMEM media (Invitrogen) with 10% of fetal bovine serum following standard protocols. For knockdown experiments 2×10^4^ (RO) or 3.5×10^4^ (T98G or U87MG) cells were transfected at the third passage with the specified double stranded RNA oligonucleotides (Stealth siRNAs, Invitrogen) and RNAiMax Lipofectamine (Invitrogen) following the reverse transfection protocol indicated in the instruction’s manual. The sequences of siRNAs utilized were: siHJURP-s: 5′ CAGGCUGAGUUUACCUUCCAGCAAA 3′, siHJURP-as: 5′ UUUGCUGGAAGGUAAACUCAGCCUG 3′, siControl-s: 5′ GCGCGCUUUGUAGGAUUCGTT 3′, siControl-as: 5′ AACGAATCCTACAAAGCGCGC 3′. Specific silencing of HJURP expression was confirmed by quantitative RT-PCR (qPCR) and western blot.

### RNA Extraction and Quantitative RT-PCR

Total RNA from tissue samples was isolated using TRIzol Reagent (Invitrogen) following the manufacturer’s instructions with an additional phenol/chlorophorm extraction to improve protein exclusion. RNA from cultured cells was extracted with the RNeasy Mini Kit (Qiagen). Purity and integrity of RNA obtained from tissue samples were evaluated as previously described [19]. cDNA synthesis was performed with the HighCapacity kit (Applied Biosystems), according to the fabricant’s recommendations, after treatment of RNA with DNAse I (Invitrogen) in the presence of RNAse inhibitor (RNAseOUT, Invitrogen). The relative mRNA expression was quantified using real-time PCR analysis in the Gene Amp® 7500 Sequence Detection System (PE Applied Biosystems). Amplification products were detected with the SYBR Green PCR Master Mix (PE Applied Biosystems). The 2^−ΔΔCT^ equation [27] was applied to calculate the relative quantities of HJURP mRNA. Mean C_T_ of non-neoplastic brain tissues was used as the calibrator sample for comparisons between tumor samples and non-neoplasic brain tissues. We utilized normalization factors calculated by geNorm software [28] on the basis of HPRT1 (Hypoxanthine guanine phosphoribosyl transferase 1) and TBP (TATA-box binding protein) expression. Primer sequences (5′ to 3′) used were HPRT-pF: TGAGGATTTGGAAAGGGTGT; HPRT-pR: GAGCACACAGAGGGCTACAA; TBP-pF: GAGCTGTGATGTGAAGTTTCC; TBP-pR: TCTGGGTTTGATCATTCTGTAG (MWG Biotech Inc); HJURP-pF: GAAGGGATGTACGTGTGACTC; HJURP-pR: CCATTCTCTGGGAGATGAAGC (Invitrogen).

### Western Blotting

For western blotting protein extracts were obtained by cell lyses directly into sample loading buffer (Laemmli loading dye, 2×). Protein extracts were separated by electrophoresis in SDS-polyacrylamide gel (10%) and western blots were performed using standard methods. The primary antibodies used were: anti-HJURP (1∶1000) and anti-tubulin mAb (1∶1000) (Sigma-Aldrich). Primary antibody against HJURP was kindly provided by Dr. Daniel R. Foltz (University of Virginia, Charlottesville, USA). Bound primary antibodies were detected by chemiluminescence (ECL kit, GE Healthcare).

### Cell Cycle, Apoptosis and Cell Death Analysis

Cell cycle analysis was performed through DNA content evaluation. Cells were harvested, washed with phosphate-buffered saline (PBS) with 5 mM EDTA, fixed in 70% ice cold ethanol for 2 hours, incubated with RNAse A (0.1 µg/µL) at 37°C for 30 minutes, labeled with propidium iodide (0.02 µg/µL) and immediately analyzed by flow cytometric analysis. Early apoptosis and effective cell death were measured by annexinV conjugated with Alexa 488 (Invitrogen) and propidium iodide labeling, respectively. After the adequate incubation periods post-transfection, cells were harvested, washed in cold PBS and diluted to 10^6^ cells/mL. To each 50 µL of cell suspension 5 µL of annexin V were added and incubated at room temperature for 15 minutes. After the incubation period, 200 µL of annexin-binding buffer were added and samples were kept on ice. Propidium iodide was added to a final concentration of 2 ng/µL and cell suspension immediately analyzed in flow cytometer (Guava easyCyte 8HT, Millipore). The results are the average of three independent experiments.

### Senescence-associated β-galactosidase Assay

For SA-β-Gal assay, cells were washed with PΒS and fixed in 4% formaldehyde dissolved in PΒS solution for 10 minutes at room temperature. After fixation, cells were washed with PBS and incubated at 37°C without CO2 with freshly prepared senescence associated (SA)-β-galactosidase staining solution: 40 mM citrate-phosphate buffer pH 6.0, 1 mg/ml 5-bromo-4-chloro-3-indolyl- β-D-galactosidase (X-Gal, Sigma), 5 mM potassium ferrocyanide and 5 mM potassium ferricyanide (Sigma), 150 mM NaCl, and 2 mM MgCl_2_ [29]. After incubation for 36 hours, SA-β-Gal-positive cells were counted under optical microscopy. The results are the average of three independent experiments.

### Public Data Banks and Statistical Analysis

The data banks used were: The Cancer Genome Atlas (TCGA), the Repository for Molecular Brain Neoplasia Data (Rembrandt) and NCBI/GEO experiments. Here, the NCBI/GEO dataset refers to the GSE4271 and GSE4412 microarray experiments, which were combined and normalized by the Robust Multi-Array Average software (Bioconductor, GCRMA package). Sample clustering showed no batch effects.

Multivariate analysis was performed using the Cox regression method [30]. To access the differences in survival based on HJURP expression, we choose the ideal cutoff in expression value by calculating ROC curves for cumulative disease or death incidence by time [31]. The cutoff for the qRT-PCR performed in-house was 39.7, and for the experiments from TCGA and NCBI/GEO were 2.4 and 6.6, respectively. Rembrandt data were analyzed with the online tool available in the repository website using the arbitrary cut off of 2 fold. The survival curves obtained for the groups with “intermediate” and “high” expression were renamed as “low-HJURP” and “high-HJURP” to be consistent with the analyses of the other data sets. For statistical evaluations we used the SPSS software version 14.0 Sciences (SPSS, Inc, Chicago, IL). Mann-Whitney *U* and Kruskal-Wallis tests were used for comparing expression level differences between the groups. Survival curves were plotted by the Kaplan-Meier method and compared by the log-rank test. To all analysis *P*<0.05 was considered statistically significant.

## Supporting Information

Figure S1 **Kaplan Meier survival curves for glioma patients according to HJURP expression using different datasets.** Rembrandt, n = 336: analysis was performed with the tool available in the repository website (https://caintegrator.nci.nih.gov/rembrandt/). GEO (GSE4271, n = 100 and GSE4412, n = 85) and TCGA, n = 424: Patients were divided in two groups of HJURP expression (low-HJURP and high-HJURP) by ROC curve analysis. The P-values shown were obtained from a long-rank test. Graphs were plotted with GraphPad Prism 4.0 software.
(TIF)

Figure S2 **Representative cell cycle distributions of RO, T98G and U87MG cells after treatment with control double-stranded siRNA (siControl) or with siRNA directed to HJURP mRNA (siHJURP).** RO, T98G and U87MG cells were transfected with siControl or siHJURP, fixed, labeled with propidium iodide (PI) and DNA content measured by flow cytometry analysis. Left panels show dot plot distribution with the cell population selected for analysis indicated (gated cells). Middle and right panels show histogram plots of cell count by PI intensity for each condition. Analysis was performed using the ModFit LT software (BD Biosciences) that is based in normally distributed Gaussian peaks.
(TIF)

Figure S3 **HJURP knockdown does not affect viability of non-tumoral human dental pulp fibroblasts (HDPC).** (A) The HJURP mRNA levels of non-tumoral HDPC transfected with control double-stranded RNA (siControl) or with siRNA directed to HJURP (siHJURP), and without treatment (WT) were determined by qRT-PCR at the seventh day after transfection. (B) Images of HDPC at the indicated times after transfection with siControl or siHJURP were captured under phase-contrast microscopy (Leica MC OS). (C) Cells subjected to the different treatments were processed for flow cytometer analysis with annexin V and propidium iodide labeling. Apoptotic or dead cells were quantified by flow cytometry (∼5,000 events) at the indicated days after transfection.
(TIF)

Figure S4 **β-Galactosidase senescence assay for RO, T98G and U87MG cells after treatment with control double-stranded siRNA (siControl), with siRNA directed against HJURP mRNA (siHJURP), or without transfection (WT).** Cells subjected to the different treatments were processed for the β-galactosidase assay at the fifth day after transfection. Images were captured under phase-contrast microscopy (Leica MC OS).
(TIF)

Table S1 **HJURP expression in tissue samples and clinical information of glioma patients.**
(XLS)

Table S2 **Percentage of senescent cells quantified by β-galactosidase assay.**
(XLS)

## References

[1] Louis DN (2006) Molecular pathology of malignant gliomas. Annu Rev Pathol 1: 97–117. 18039109 10.1146/annurev.pathol.1.110304.100043

[2] Louis DN, Ohgaki H, Wiestler OD, Cavenee WK, Burger PC, et al (2007) The 2007 WHO classification of tumours of the central nervous system. Acta Neuropathol 114: 97–109. 17618441 10.1007/s00401-007-0243-4 PMC1929165

[3] Behin A, Hoang-Xuan K, Carpentier AF, Delattre JY (2003) Primary brain tumours in adults. Lancet 361: 323–331. 12559880 10.1016/S0140-6736(03)12328-8

[4] Phillips HS, Kharbanda S, Chen R, Forrest WF, Soriano RH, et al (2006) Molecular subclasses of high-grade glioma predict prognosis, delineate a pattern of disease progression, and resemble stages in neurogenesis. Cancer Cell 9: 157–173. 16530701 10.1016/j.ccr.2006.02.019

[5] Li A, Walling J, Ahn S, Kotliarov Y, Su Q, et al (2009) Unsupervised analysis of transcriptomic profiles reveals six glioma subtypes. Cancer Res 69: 2091–2099. 19244127 10.1158/0008-5472.CAN-08-2100 PMC2845963

[6] Liang Y, Diehn M, Watson N, Bollen AW, Aldape KD, et al (2005) Gene expression profiling reveals molecularly and clinically distinct subtypes of glioblastoma multiforme. Proc Natl Acad Sci U S A 102: 5814–5819. 15827123 10.1073/pnas.0402870102 PMC556127

[7] Petalidis LP, Oulas A, Backlund M, Wayland MT, Liu L, et al (2008) Improved grading and survival prediction of human astrocytic brain tumors by artificial neural network analysis of gene expression microarray data. Mol Cancer Ther 7: 1013–1024. 18445660 10.1158/1535-7163.MCT-07-0177 PMC2819720

[8] Verhaak RG, Hoadley KA, Purdom E, Wang V, Qi Y, et al (2010) Integrated genomic analysis identifies clinically relevant subtypes of glioblastoma characterized by abnormalities in PDGFRA, IDH1, EGFR, and NF1. Cancer Cell 17: 98–110. 20129251 10.1016/j.ccr.2009.12.020 PMC2818769

[9] Hegi ME, Diserens AC, Gorlia T, Hamou MF, de Tribolet N, et al (2005) MGMT gene silencing and benefit from temozolomide in glioblastoma. N Engl J Med 352: 997–1003. 15758010 10.1056/NEJMoa043331

[10] Parsons DW, Jones S, Zhang X, Lin JC, Leary RJ, et al (2008) An integrated genomic analysis of human glioblastoma multiforme. Science 321: 1807–1812. 18772396 10.1126/science.1164382 PMC2820389

[11] Yan H, Parsons DW, Jin G, McLendon R, Rasheed BA, et al (2009) IDH1 and IDH2 mutations in gliomas. N Engl J Med 360: 765–773. 19228619 10.1056/NEJMoa0808710 PMC2820383

[12] de Tayrac M, Aubry M, Saikali S, Etcheverry A, Surbled C, et al (2011) A 4-gene signature associated with clinical outcome in high-grade gliomas. Clin Cancer Res 17: 317–327. 21224364 10.1158/1078-0432.CCR-10-1126

[13] Foltz DR, Jansen LE, Bailey AO, Yates JR, 3rd, Bassett EA, et al (2009) Centromere-specific assembly of CENP-a nucleosomes is mediated by HJURP. Cell 137: 472–484. 19410544 10.1016/j.cell.2009.02.039 PMC2747366

[14] Dunleavy EM, Roche D, Tagami H, Lacoste N, Ray-Gallet D, et al (2009) HJURP is a cell-cycle-dependent maintenance and deposition factor of CENP-A at centromeres. Cell 137: 485–497. 19410545 10.1016/j.cell.2009.02.040

[15] Barnhart MC, Kuich PH, Stellfox ME, Ward JA, Bassett EA, et al (2011) HJURP is a CENP-A chromatin assembly factor sufficient to form a functional de novo kinetochore. J Cell Biol 194: 229–243. 21768289 10.1083/jcb.201012017 PMC3144403

[16] Kato T, Sato N, Hayama S, Yamabuki T, Ito T, et al (2007) Activation of Holliday junction recognizing protein involved in the chromosomal stability and immortality of cancer cells. Cancer Res 67: 8544–8553. 17823411 10.1158/0008-5472.CAN-07-1307

[17] Mishra PK, Au WC, Choy JS, Kuich PH, Baker RE, et al (2011) Misregulation of Scm3p/HJURP Causes Chromosome Instability in Saccharomyces cerevisiae and Human Cells. PLoS Genet 7: e1002303. 21980305 10.1371/journal.pgen.1002303 PMC3183075

[18] Hu Z, Huang G, Sadanandam A, Gu S, Lenburg ME, et al (2010) The expression level of HJURP has an independent prognostic impact and predicts the sensitivity to radiotherapy in breast cancer. Breast Cancer Res 12: R18. 20211017 10.1186/bcr2487 PMC2879562

[19] Valente V, Teixeira SA, Neder L, Okamoto OK, Oba-Shinjo SM, et al (2009) Selection of suitable housekeeping genes for expression analysis in glioblastoma using quantitative RT-PCR. BMC Mol Biol 10: 17. 19257903 10.1186/1471-2199-10-17 PMC2661085

[20] Roy K, Wang L, Makrigiorgos GM, Price BD (2006) Methylation of the ATM promoter in glioma cells alters ionizing radiation sensitivity. Biochem Biophys Res Commun 344: 821–826. 16631604 10.1016/j.bbrc.2006.03.222

[21] Maehara K, Takahashi K, Saitoh S (2010) CENP-A reduction induces a p53-dependent cellular senescence response to protect cells from executing defective mitoses. Molecular and cellular biology 30: 2090–2104. 20160010 10.1128/MCB.01318-09 PMC2863584

[22] Heo JI, Cho JH, Kim JR (2013) HJURP Regulates Cellular Senescence in Human Fibroblasts and Endothelial Cells Via a p53-Dependent Pathway. The journals of gerontology Series A, Biological sciences and medical sciences. 10.1093/gerona/gls257 23292286

[23] Clark MJ, Homer N, O’Connor BD, Chen Z, Eskin A, et al (2010) U87MG decoded: the genomic sequence of a cytogenetically aberrant human cancer cell line. PLoS Genet 6: e1000832. 20126413 10.1371/journal.pgen.1000832 PMC2813426

[24] Pleasance ED, Cheetham RK, Stephens PJ, McBride DJ, Humphray SJ, et al (2010) A comprehensive catalogue of somatic mutations from a human cancer genome. Nature 463: 191–196. 20016485 10.1038/nature08658 PMC3145108

[25] Stephens PJ, McBride DJ, Lin ML, Varela I, Pleasance ED, et al (2009) Complex landscapes of somatic rearrangement in human breast cancer genomes. Nature 462: 1005–1010. 20033038 10.1038/nature08645 PMC3398135

[26] Galante PA, Parmigiani RB, Zhao Q, Caballero OL, de Souza JE, et al (2011) Distinct patterns of somatic alterations in a lymphoblastoid and a tumor genome derived from the same individual. Nucleic Acids Res 39: 6056–6068. 21493686 10.1093/nar/gkr221 PMC3152357

[27] Livak KJ, Schmittgen TD (2001) Analysis of relative gene expression data using real-time quantitative PCR and the 2(-Delta Delta C(T)) Method. Methods 25: 402–408. 11846609 10.1006/meth.2001.1262

[28] Vandesompele J, De Preter K, Pattyn F, Poppe B, Van Roy N, et al (2002) Accurate normalization of real-time quantitative RT-PCR data by geometric averaging of multiple internal control genes. Genome Biol 3: RESEARCH0034. 12184808 10.1186/gb-2002-3-7-research0034 PMC126239

[29] Dimri GP, Lee X, Basile G, Acosta M, Scott G, et al (1995) A biomarker that identifies senescent human cells in culture and in aging skin in vivo. Proc Natl Acad Sci U S A 92: 9363–9367. 7568133 10.1073/pnas.92.20.9363 PMC40985

[30] Cox DR, McCullagh P (1982) Some aspects of analysis of covariance. Biometrics 38: 541–561. 7171689

[31] Heagerty PJ, Lumley T, Pepe MS (2000) Time-dependent ROC curves for censored survival data and a diagnostic marker. Biometrics 56: 337–344. 10877287 10.1111/j.0006-341x.2000.00337.x

