# Supplementary figures and images for: Modulation of HJURP (Holliday Junction-Recognizing Protein) Levels Is Correlated with Glioblastoma Cells Survival
Source: PLoS One. 2013 Apr 25;8(4):e62200. doi: 10.1371/journal.pone.0062200 (PMC3636219; doi:10.1371/journal.pone.0062200)

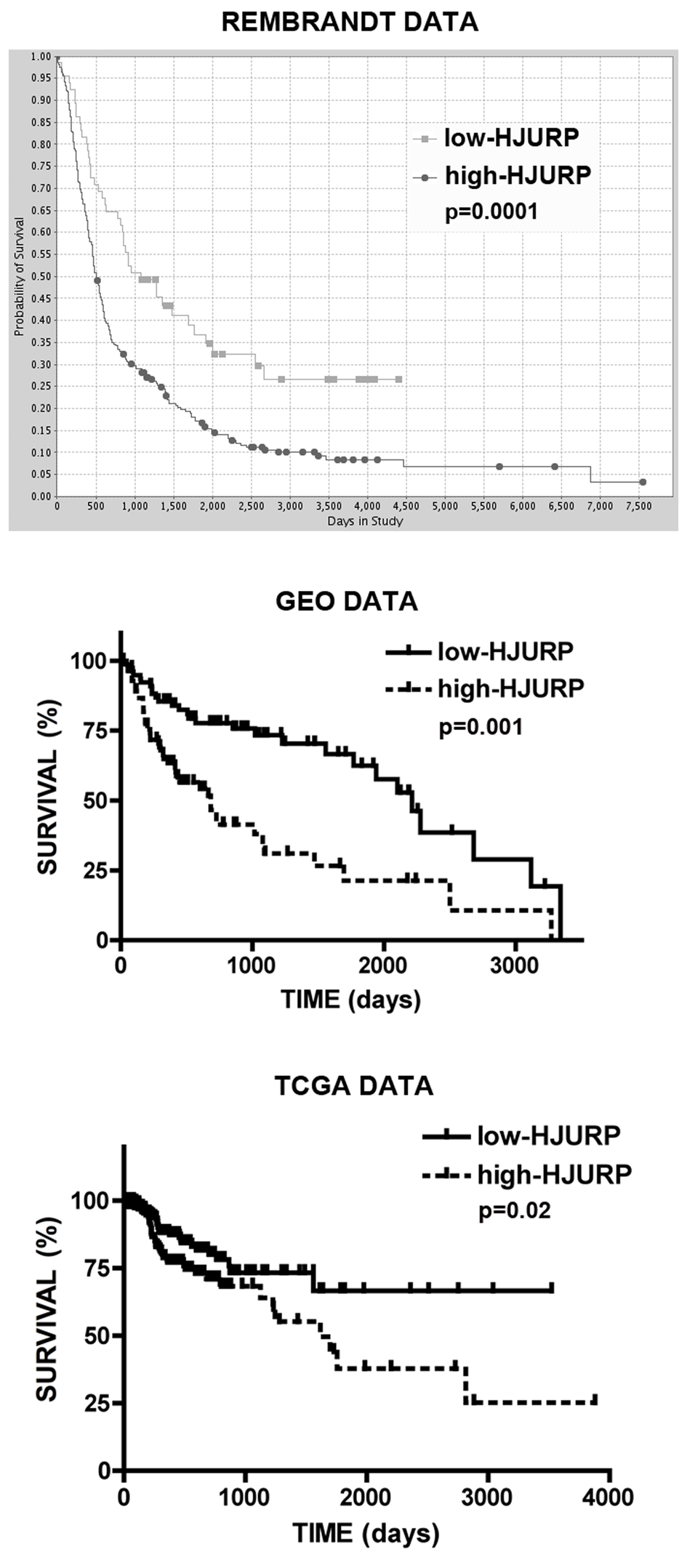

Supplement: Figure S1 — Kaplan Meier survival curves for glioma patients according to HJURP expression using different datasets. Rembrandt, n = 336: analysis was performed with the tool available in the repository website (https://caintegrator.nci.nih.gov/rembrandt/). GEO (GSE4271, n = 100 and GSE4412, n = 85) and TCGA, n = 424: Patients were divided in two groups of HJURP expression (low-HJURP and high-HJURP) by ROC curve analysis. The P-values shown were obtained from a long-rank test. Graphs were plotted with GraphPad Prism 4.0 software. (TIF) [file pone.0062200.s001.tif]

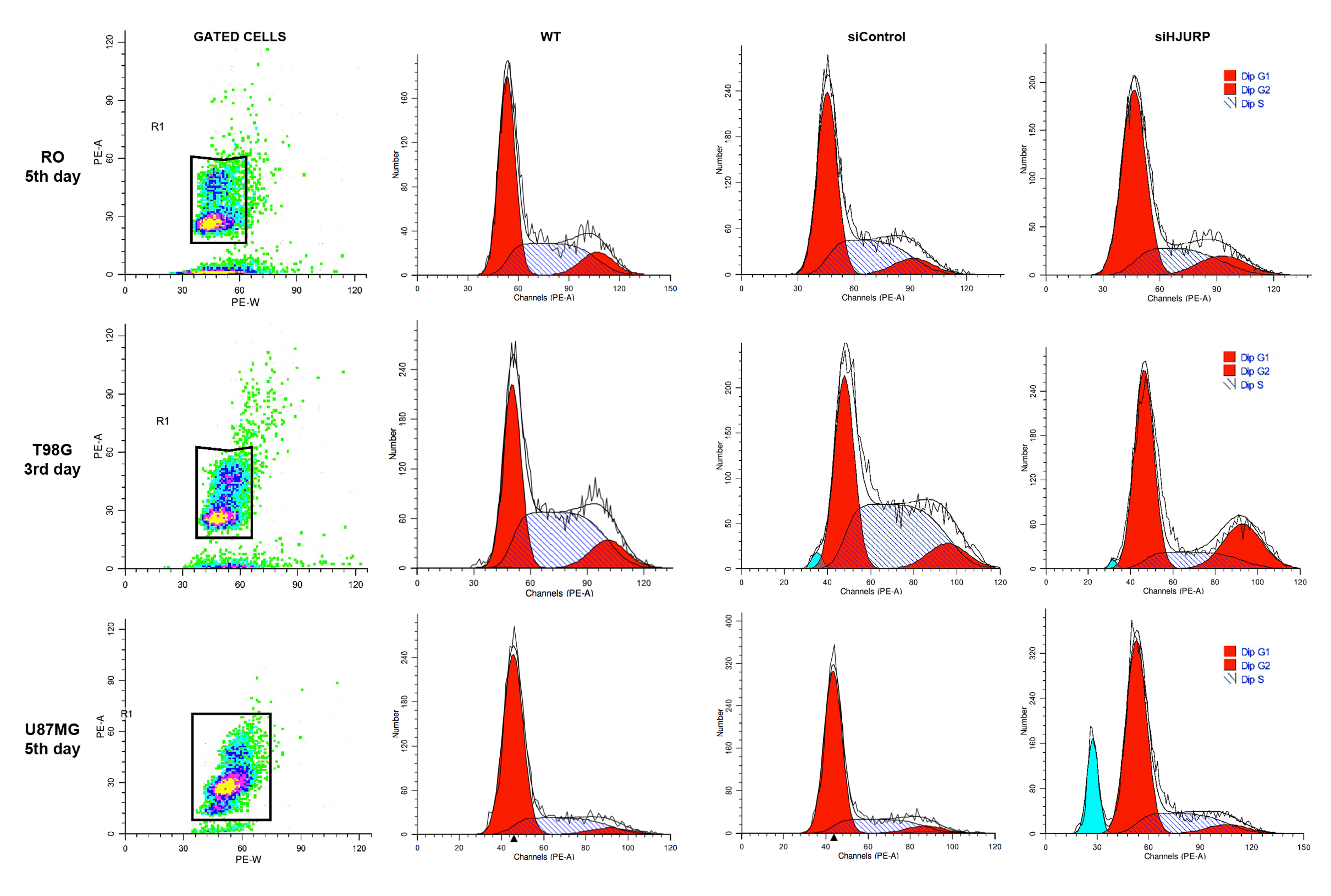

Supplement: Figure S2 — Representative cell cycle distributions of RO, T98G and U87MG cells after treatment with control double-stranded siRNA (siControl) or with siRNA directed to HJURP mRNA (siHJURP). RO, T98G and U87MG cells were transfected with siControl or siHJURP, fixed, labeled with propidium iodide (PI) and DNA content measured by flow cytometry analysis. Left panels show dot plot distribution with the cell population selected for analysis indicated (gated cells). Middle and right panels show histogram plots of cell count by PI intensity for each condition. Analysis was performed using the ModFit LT software (BD Biosciences) that is based in normally distributed Gaussian peaks. (TIF) [file pone.0062200.s002.tif]

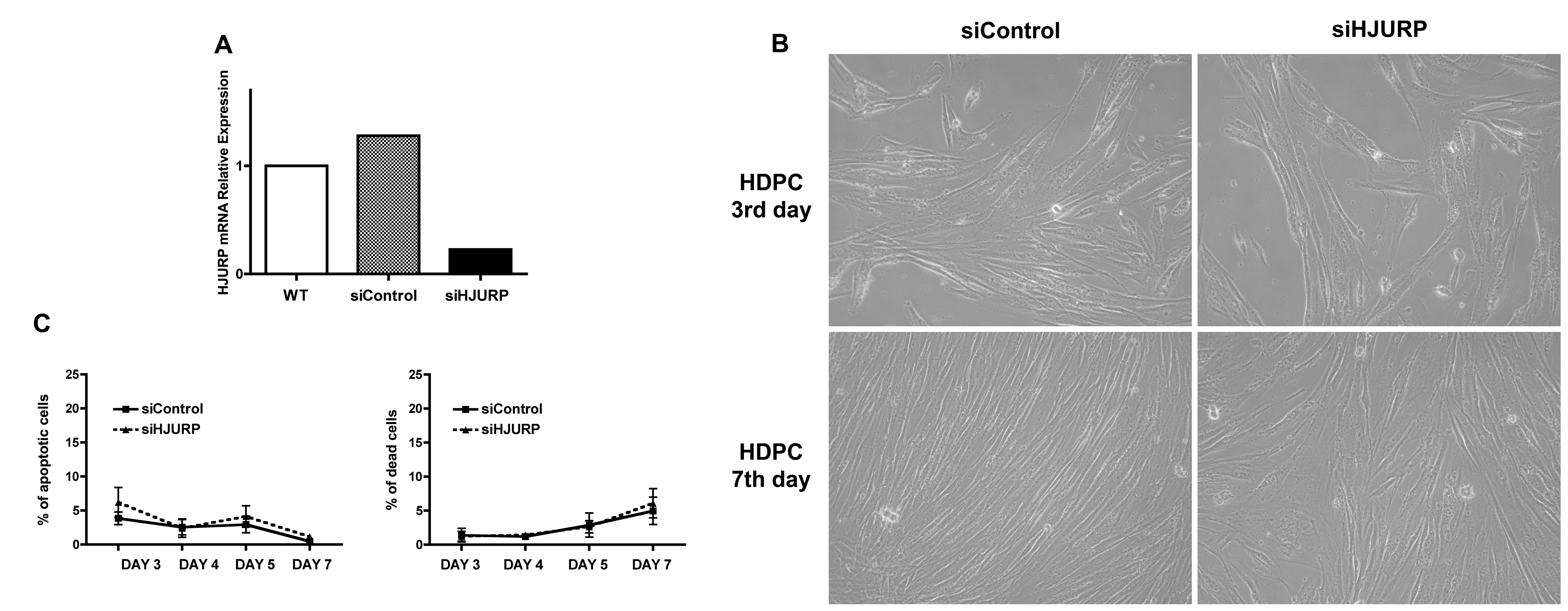

Supplement: Figure S3 — HJURP knockdown does not affect viability of non-tumoral human dental pulp fibroblasts (HDPC). (A) The HJURP mRNA levels of non-tumoral HDPC transfected with control double-stranded RNA (siControl) or with siRNA directed to HJURP (siHJURP), and without treatment (WT) were determined by qRT-PCR at the seventh day after transfection. (B) Images of HDPC at the indicated times after transfection with siControl or siHJURP were captured under phase-contrast microscopy (Leica MC OS). (C) Cells subjected to the different treatments were processed for flow cytometer analysis with annexin V and propidium iodide labeling. Apoptotic or dead cells were quantified by flow cytometry (∼5,000 events) at the indicated days after transfection. (TIF) [file pone.0062200.s003.tif]

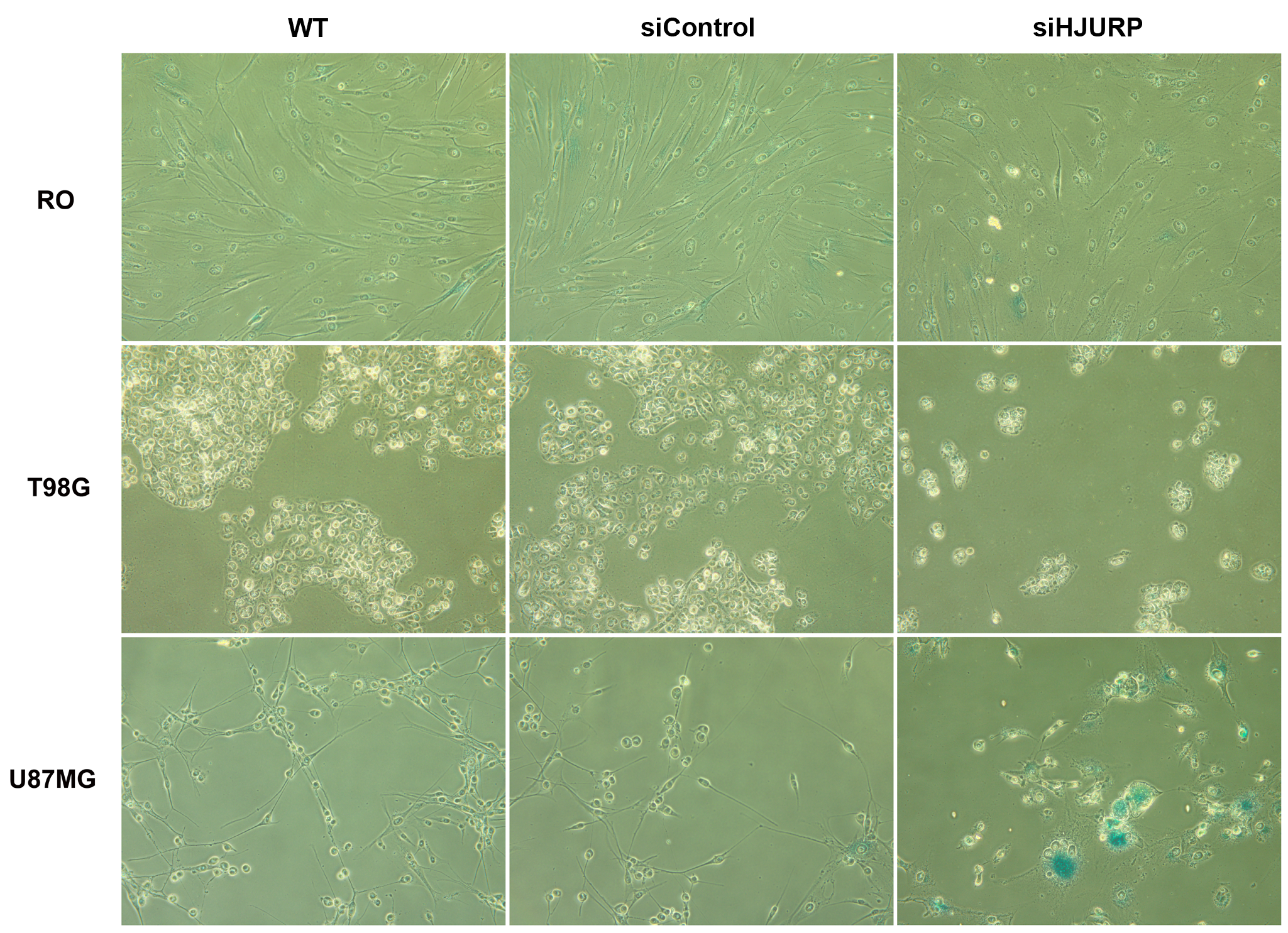

Supplement: Figure S4 — β-Galactosidase senescence assay for RO, T98G and U87MG cells after treatment with control double-stranded siRNA (siControl), with siRNA directed against HJURP mRNA (siHJURP), or without transfection (WT). Cells subjected to the different treatments were processed for the β-galactosidase assay at the fifth day after transfection. Images were captured under phase-contrast microscopy (Leica MC OS). (TIF) [file pone.0062200.s004.tif]
